# Case report: A rare case of renal tuberculosis combined with bladder cancer

**DOI:** 10.3389/fonc.2024.1423744

**Published:** 2024-11-26

**Authors:** Yang Xiang, Zhou Wen, Meng Yang, Han Lyu, Zongyu Chen, Dongbo Yuan, Jianguo Zhu

**Affiliations:** ^1^ Department of Urology, Guizhou Provincial People's Hospital, Guiyang, China; ^2^ School of the First Clinical Medicine, Zunyi Medical University, Zunyi, China; ^3^ School of the First Clinical Medicine, Guizhou Medical University, Guiyang, China

**Keywords:** renal tuberculosis, autonephrectomy, urinary tuberculosis, bladder cancer, BCG, case report

## Abstract

We report a case of renal tuberculosis combined with bladder cancer. The patient was a 57-year-old man with no history of tuberculosis who presented with hematuria and signs of urinary tract irritation. Computed tomography (CT) showed florid, bowel-filling calcifications at the level of the right renal hilum, multiple hyperdense shadows from the right renal pelvis to the ureter, and left pyelo-ureteral effusion. Enhanced CT showed localized protrusion and marked enhancement of bladder tissue. Blood TSPOT.TB was positive, erythrocyte sedimentation rate (ESR) was increased, and urine Mycobacterium tuberculosis DNA was negative. Cystoscopy showed an irregular bulge at the bladder neck opening, and pathological examination diagnosed high-grade papillary carcinoma of the uroepithelium. The diagnosis was tuberculosis of the right kidney and bladder tumor. The patient was re-visited due to anuria for 2 days and underwent emergency left percutaneous nephrostomy (PCN) to improve the left kidney function. Given the potential for extensive infiltration into the muscular layer of the bladder tumor, a transurethral resection of the bladder tumor (TURBT) was advocated as an initial step to obtain tissue for diagnostic confirmation. Following this, a right nephrectomy and radical cystectomy to address the bladder cancer would have been performed. Nevertheless, the patient declined surgery due to the associated risks and succumbed to the illness 7 months post-follow-up. This is a rare case, and informed consent was obtained from the patient and her family.

## Case report

**Figure 1:**
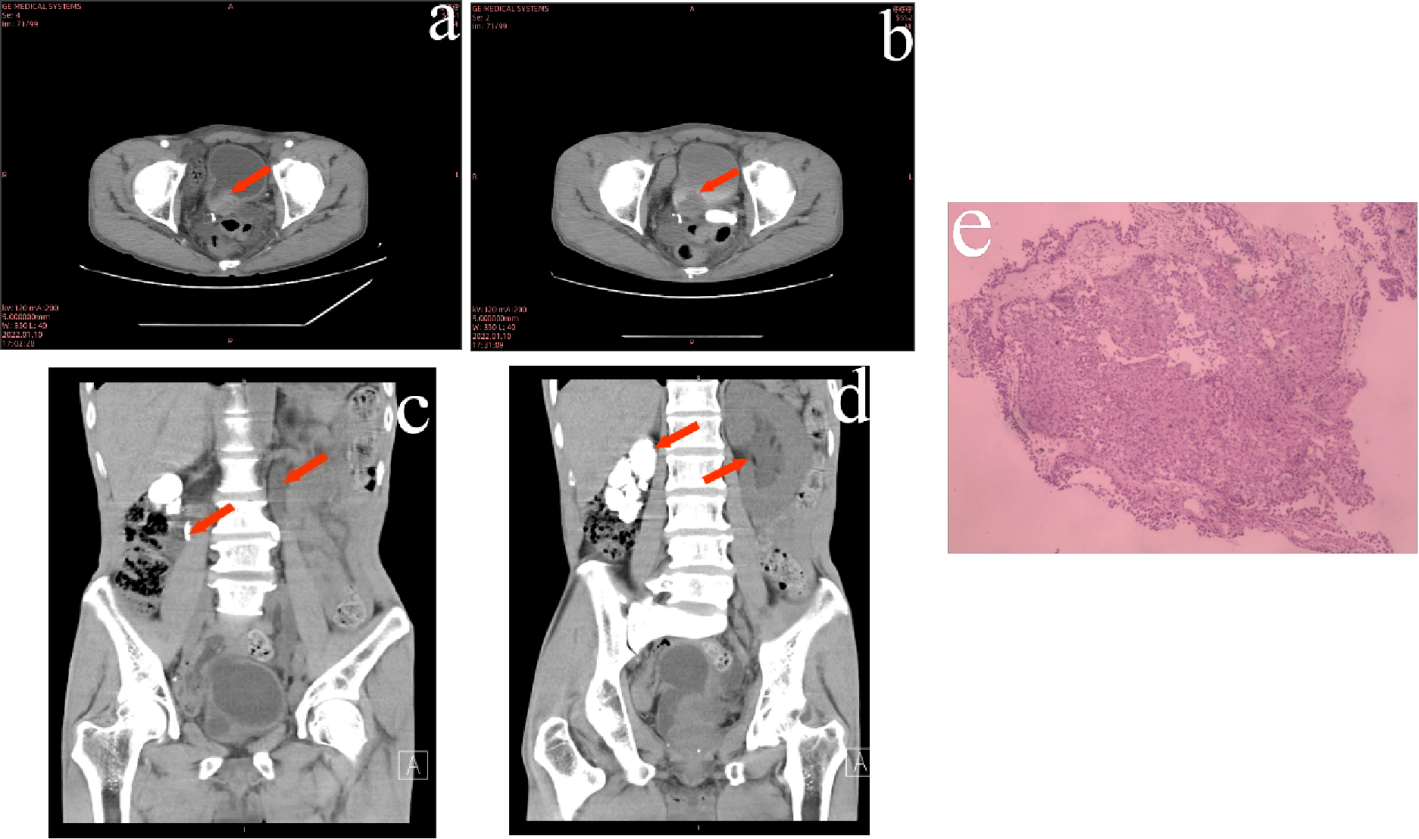
**(A)** Enhanced CT showed markedly enhanced signal of the protruding tissue in the bladder. **(B)** Intravesical filling defects in the bladder seen during the excretory phase of enhanced CT. **(C, D)** CT showed a filled bowel lumen-like calcification at the level of the right renal hilum, left hydronephrosis and ureteral effusion. **(E)** Histopathological biopsy of the bladder suggested high-grade papillary uroepithelial carcinoma. (Hematoxylin-eosin staining).

Male, 57 years old, consulted for “Hematuria for 3 years, aggravated for 6 months”. The hematuria was continuous with the presence of small blood clots. Concurrent symptoms including frequent urination, urgency, and painful urination developed 6 months prior. No fever, night sweats, or history of tuberculosis was reported. The patient also exhibited anorexia, lethargy, and a weight loss of 10 kg over the past 6 months. CT imaging revealed irregular bladder morphology, soft tissue thickening at the base, localized protrusion, and significant enhancement (**Figure 1A**), and obvious filling-defect in the excretory phase (**Figure 1B**). CT scans also disclosed a reduction in the volume of the right kidney, multiple hyperdense shadows from the renal pelvis to the ureter on the right, and left hydronephrosis with complete left hydroureterosis (**Figures 1C, D**). Cystoscopy showed that the bladder neck orifice was not smooth, and irregular redundant organisms were seen in the direction of 4-7 o’clock, about 1.0×0. 8 cm, and bilateral ureteral orifices could not be seen, and the pathological biopsy of the redundant organisms at the bladder neck orifice suggested high-grade papillary carcinoma of the uroepithelium, which did not obtain the muscularis propria, and did not exclude the possibility of infiltration (**Figure 1E**). Urinalysis revealed an erythrocyte count of 22,770 cells/µl, a leukocyte count of 216 cells/µl, and a hemoglobin level of 70 g/L. The blood TSPOT.TB test was positive, the ESR was 28 mm/h. Urinary Mycobacterium tuberculosis DNA testing was negative. Isoniazid, rifampicin, pyrazinamide and ethambutol were proposed as anti-tuberculosis treatment for 2 weeks followed by surgery. The patient was re-visited for “no urine for 2 days”, with a blood potassium of 6.60 mmol/L, a serum creatinine (SCr) of 833 μmol/L, and an estimated glomerular filtration rate (eGFR) of 6 ml/min/1.73 m^2^. The diagnosis was established as follows: 1) Acute left renal insufficiency; 2) High-grade bladder urothelial carcinoma; 3) Right autonephrectomyn. Management involved potassium-lowering treatment and urgent left PCN, which drained approximately 1,200 ml of urine per day. SCr, serum potassium and eGFR, returned to normal after 3 days.

CT enhancement scans and pathological analysis suggested a high probability of infiltration in the patient’s bladder tumor. It was prepared to perform another biopsy to clarify the tumor infiltration after the patient’s condition was stabilized. However, the patient and his family refused further treatment. The patient’s left nephrostomy tube was changed monthly. Seven months later, the patient passed away due to the worsening of his condition.

Renal tuberculosis was diagnosed based on the characteristic CT findings (bowel lumen-like calcification at the renal hilum, urothelial calcification, and contralateral renal pelvis or ureteral effusion), a positive blood TSPOT.TB test, and elevated blood sedimentation rates. Despite refusing surgery due to the surgery risks, the patient was unable to provide a renal biopsy sample, precluding a definitive pathological diagnosis.

## Discussion

Bacillus Calmette-Guérin (BCG) was an attenuated bovine strain of Mycobacterium tuberculosis and was currently the most effective postoperative treatment for non-muscle invasive bladder cancer (NMIBC) (1). The co-occurrence of renal tuberculosis and bladder cancer was exceptionally rare in clinical practice. The simultaneous presence of bladder cancer with renal tuberculosis in this instance was further anomalies. We propose several potential factors that could explain the concurrent presence of these conditions: first, individual variation may result in the tumor cells of this patient failing to adequately respond to Mycobacterium tuberculosis, thereby enabling the tumor to further progress (2). Second, tumor development likely occurred subsequent to renal self-interception, with urine containing Mycobacterium tuberculosis flowing into the bladder in reduced numbers or even absent, thus insufficient to stimulate a local inflammatory response capable of eliminating the tumor cells (3, 4). Lastly, the high bladder tumor load and relatively low Mycobacterium tuberculosis count further diminished the potential of effective anti-tumor activity by the bacteria (5).

BCG was considered the most effective treatment for high-risk NMIBC, yet a significant number of patients relapse following BCG bladder instillation. Recognizing the degree of differentiation and complexity of bladder tumors, conducting comprehensive molecular studies on cases of urothelial tuberculosis concurrent with bladder cancer could potentially reveal specific markers for these tumors. Such findings might further elucidate the resistance mechanisms in some bladder cancers to BCG instillation, potentially leading to novel treatment strategies to combat BCG resistance.

The likelihood of renal tuberculosis co-occurring with bladder cancer is remarkably low but does exist, necessitating clinical awareness to avoid underdiagnosis and to address appropriate treatment modalities for these cases. Given that the biopsy did not include muscle tissue but is based on an enhanced CT scan, the risk of infiltration is considered high. Consequently, a further biopsy is planned to confirm the diagnosis. In instances of non-functioning kidneys accompanied by MIBC, both nephrectomy and radical bladder resection are recommended. However, both renal tuberculosis and bladder tumor are chronic, consumptive conditions associated with anemia from prolonged tumor bleeding. The risk of surgery is significant when both procedures are done concurrently. The authors suggest that TB nephrectomy can be performed initially, followed by GC chemotherapy regimen to decrease the tumor burden in the bladder. Subsequently, the second stage could involve radical cystectomy. Alternatively, concurrent anti-tuberculosis treatment and chemotherapy can be initiated, after which the patient may undergo simultaneous TB nephrectomy and cystectomy when conditionally stable.

## Data Availability

The original contributions presented in the study are included in the article/**Supplementary Material**. Further inquiries can be directed to the corresponding author.
